# Supplementary material for: Establishment of Stem Cell-like Cells of Sida hermaphrodita (L.) Rusby from Explants Containing Cambial Meristems
Source: Int J Mol Sci. 2022 Jul 11;23(14):7644. doi: 10.3390/ijms23147644 (PMC9320681; doi:10.3390/ijms23147644)
Supplement: Supplementary file 1 [file ijms-23-07644-s001.zip › ijms-1787162-supplementary.pdf]

Table S1. Effects of explant types and plant growth hormones and regulators for induction and growth of callus biomass of *S. hermaphrodita*.

Explant type	Plant growth regulators		Callus induction [%]		Callus weight [g]		Callus structure		Colour of callus	
	Auxin [1 mg/L]	Cytokinin [0.5 mg/L]	4 weeks	16 weeks	4 weeks	16 weeks	4 weeks	16 weeks	4 weeks	16 weeks
Leaf	2.4-D	-	4	8	0.018 ± 0.008^{CD}	0.128 ± 0.036^{AB}	C	C	white	white, light orange/yellow
		KIN	100	100	0.069 ± 0.035^{BCD}	0.680 ± 0.466^{A}	C	C	white, orange	white, yellow, orange, brown
		BAP	12	32	0.024 ± 0.013^{CD}	0.468 ± 0.377^{AB}	C	C	white	white, light yellow, orange
		2ip	20	36	0.069 ± 0.054^{BCD}	0.323 ± 0.193 ^{AB}	C	C	white	white, light yellow/orange
	NAA	KIN	12	28	0.071 ± 0.035^{BCD}	0.308 ± 0.391 ^{AB}	C	C	orange	white, orange, brown, black
		BAP	0	36	0.166 ± 0.205^{AB}	0.465 ± 0.492 ^{AB}	-	C	-	white, orange, yellow
		2ip	0	8	0.030 ± 0.007^{CD}	0.074 ± 0.014^{B}	-	C	-	yellow, white
	IBA	KIN	0	8	0.010 ± 0.010^{D}	0.085 ± 0.025^{B}	-	C	-	orange, yellow
		BAP	24	48	0.123 ± 0.095^{ABC}	0.702 ±1.139^{A}	C	C	orange, yellow	white, orange, yellow, brown, black
		2ip	12	13	0.212 ± 0.128^{A}	0.234 ± 0.101 ^{AB}	C	C	orange, white	white, orange
Stem	2.4-D	-	96	100	0.225 ± 0.063^{C}	0.508 ± 0.057^{FG}	WS	WS	white, light yellow/green	white, green, yellow, brown, orange
		KIN	72	100	0.490 ± 0.230^{A}	7.244 ± 3.273^{AB}	C	C	white, light yellow	light yellow, white, orange, brown
		BAP	60	96	0.290 ± 0.051^{BC}	4.372 ± 0.773^{CD}	C	C	white, ligt yellow/green	white, yellow, orange, brown, black
		2ip	80	96	0.348 ± 0.067^{ABC}	1.3 ± 0.497^{EFG}	WS	WSF	white, ligt yellow/green	white, green, yellow, brown
	NAA	KIN	90	95	0.213 ± 0.141^{CD}	3.109 ± 2.943^{CDE}	C	WS	white, light green	white, yellow, orange, brown, black
		BAP	90	100	0.458 ± 0.200^{AB}	4.958 ± 1.272^{BC}	C	C	white, light yellow/green	white, yellow, brown, orange, green, black
		2ip	85	85	0.292 ± 0.204^{BC}	2.610 ± 2.206^{DEF}	C	WS	white, light yellow	white, yellow, orange, brown
	IBA	KIN	25	80	0.042 ± 0.015^{D}	0.153 ± 0.056^{G}	C	C	white	white, yellow, orange
		BAP	100	100	0.265 ± 0.071^{C}	8.973 ± 2.389^{A}	C	C	white	yellow, white, orange
		2ip	68	68	0.205 ± 0.105^{CD}	0.585 ± 0.172^{FG}	WS	C	light yellow	brown, yellow, green

Table S1. *Cont.*

Explant type	Plant growth regulators		Callus induction [%]		Callus weight [g]		Callus structure		Colour of callus	
	Auxin [1 mg/L]	Cytokinin [0.5 mg/L]	4 weeks	16 weeks	4 weeks	16 weeks	4 weeks	16 weeks	4 weeks	16 weeks
Petiole	2.4-D	-	92	96	0.098 ± 0.035^{A}	0.325 ± 0.108^{CD}	WS	WS	light yellow/green	light yellow
		KIN	60	96	0.046 ± 0.033^{B}	3.119 ± 1.652^{A}	C	WS	white, light yellow	white, orange, brown, light yellow
		BAP	32	80	0.032 ± 0.025^{B}	1.456 ± 0.870^{B}	C	WS	white, light yellow	white, light yellow, black
		2ip	76	84	0.128 ± 0.088^{A}	0.477 ± 0.172^{CD}	C	WSF	yellow, white, orange	yellow, white, orange
	NAA	KIN	32	81	0.033 ± 0.013^{B}	0.588 ± 0.422^{BCD}	C	C	white, light yellow	yellow, white, orange
		BAP	56	75	0.050 ± 0.018^{B}	1.119 ± 0.741^{BC}	C	C	light yellow	yellow, orange, white
		2ip	25	25	0.025 ± 0.016^{B}	0.194 ± 0.253^{CD}	C	C	white, light yellow/green	yellow
	IBA	KIN	56	100	0.024 ± 0.009^{B}	0.206 ± 0.055^{CD}	C	C	white	white, yellow, orange
		BAP	32	44	0.028 ± 0.019^{B}	0.766 ± 1.071^{BCD}	C	C	white, light yellow	yellow, brown, orange
		2ip	0	12	0.024 ± 0.019^{B}	0.160 ± 0.123^{D}	-	C	-	orange, yellow
Root	2.4-D	-	84	90	0.284 ± 0.153^{A}	0.727 ± 0.241^{B}	C	WSF	white, light yellow	white, light yellow
		KIN	44	100	0.184 ± 0.156^{AB}	0.926 ± 0.347^{B}	C	WSF	white, light yellow	yellow, brown
		BAP	32	100	0.127 ± 0.069^{B}	2.294 ± 1.620^{A}	C	WS	white, light yellow	yellow, black
		2ip	44	92	0.279 ± 0.131^{A}	0.494 ± 0.118^{B}	C	WSF	white, light yellow	white, light yellow
	NAA	KIN	50	85	0.179 ± 0.077^{AB}	1.016 ± 0.565^{B}	C	WS	white, light yellow	yellow, orange, white
		BAP	75	100	0.124 ± 0.057^{B}	2.658 ± 0.438^{A}	C	WS	white, light yellow	yellow, orange, green
		2ip	85	95	0.201 ± 0.073^{AB}	0.365 ± 0.101^{B}	C	WS	white, light yellow	yellow, orange
	IBA	KIN	56	100	0.098 ± 0.058^{B}	1.116 ± 1.041^{B}	C	C	light yellow	yellow, orange, brown
		BAP	20	65	0.137 ± 0.073^{B}	0.592 ± 0.433^{B}	C	C	light yellow	yellow
		2ip	100	100	0.191 ± 0.097^{AB}	0.551 ± 0.194^{B}	C	C/WS	brown, light yellow	brown, yellow, orange

Callus structre: C - compact, WS - watery soft, WSF - watery soft and friable. Exponents ^{A,B,C,D,E,F,G} indicate significant different average values ($p<0.05$). 2,4-D – 2,4-dichlorophenoxyacetic acid, NAA – 1-naphthylacetic acid, IBA – indole-3-butyric acid, 2ip – 6-(γ,γ-dimethylallylamino)purine, KIN – kinetin, BAP – 6-benzylaminopurine.
